# Clinical Efficacy and Safety of transarterial chemoembolization Combined with Targeted Therapy for primary hepatocellular carcinoma

**DOI:** 10.12669/pjms.40.8.8982

**Published:** 2024-09

**Authors:** Xiao Wang, Dan Zhang, Kang Li, Peng Guo, Zhi-xiong Lei

**Affiliations:** 1 Xiao Wang, Department of Infectious Diseases, Renmin Hospital, Hubei University of Medicine, Shiyan 442000, Hubei, P.R. China; 2 Dan Zhang, Department of Infectious Diseases, Renmin Hospital, Hubei University of Medicine, Shiyan 442000, Hubei, P.R. China; 3 Kang Li, Department of Nursing, National Medicine Dongfeng General Hospital, Hubei University of Medicine, Shiyan 442000, Hubei, P.R. China; 4 Peng Guo, Department of Infectious Diseases, Renmin Hospital, Hubei University of Medicine, Shiyan 442000, Hubei, P.R. China; 5 Zhi-xiong Lei, Department of Infectious Diseases, Renmin Hospital, Hubei University of Medicine, Shiyan 442000, Hubei, P.R. China

**Keywords:** TACE, Targeted therapy, PHC, Clinical efficacy, Safety

## Abstract

**Objective:**

To explore the clinical efficacy and safety of transarterial chemoembolization (TACE) combined with targeted therapy for primary hepatocellular carcinoma (PHC).

**Methods:**

This was a retrospective study. Retrospective selection of 150 PHC patients admitted to the Renmin Hospital, Hubei University of Medicine January 2019 and June 2021 were included. The patients were divided into the control group and the experimental group according to their treatment regimens. The control group received TACE treatment, while the experimental group received TACE combined with targeted therapy. We analyze the relevant data of two groups of patients and evaluate the clinical efficacy and safety of TACE combined with targeted therapy.

**Results:**

The tumor remission rate and control rate in the control group were 41.89% and 75.68%, respectively, while those in the experimental group were 77.63% and 90.79%, with statistically significant differences (p<0.05). The 1-year and 3-year recurrence rates in the control group were 52.71% and 98.65%, respectively, while those in the experimental group were 39.47% and 61.84%, with statistically significant differences (p<0.05). After treatment, the AFP, VEGF, ALT, and AST in the experimental group were significantly reduced compared to the control group (p<0.05). During the treatment period, the incidence and severity of nausea, vomiting, and fever in the experimental group were significantly lower than those in the control group (p<0.05).

**Conclusion:**

The clinical efficacy of TACE combined with targeted therapy for PHC is superior to that of TACE alone, with improved disease control rate, improved long-term survival rate, and good safety.

## INTRODUCTION

PHC is a type of malignant tumor originating from liver epithelial tissue, mainly includes hepatocellular carcinoma (HCC), cholangiocarcinoma (CCA), and combined hepatocellular cholangiocarcinoma(cHCC). HCC is the primary type of PHC, accounting for over 90% of liver cancer. The incidence and mortality rates rank fifth and third in global malignant tumors, making it an important factor influencing the safety of human life in the world.^1^ With the development of medical technology, the early diagnosis and survival rates of PHC have been effectively improved. TACE is the main treatment for PHC, and its efficacy has been widely recognized in the clinical medicine.^2^ The therapeutic effect of single TACE is poor, and repeated multiple TACE can increase hepatic tissue hypoxia, leading to increased hepatic function damage, affecting therapeutic efficacy and leading to a poor prognosis for patients.^3^ Targeted therapy is an intervention scheme emerging in the context of the development of systemic anti-tumor therapies.^4^ Targeted therapy can inhibit vascular endothelial growth factor receptors, tumor angiogenesis, and tumor development.^5^

TACE and targeted therapy are both effective intervention regimens for PHC. However, studies have found that the clinical efficacy and safety of TACE and targeted therapy alone cannot meet the treatment needs of PHC, and the combination therapy is effective in improving the prognosis and prolonging the survival of PHC patients. Studies have found that TACE combined with targeted therapy can effectively enhance the therapeutic effect of TACE.^6^ In order to explore the safety and efficacy of TACE combined with targeted therapy in PHC treatment, PHC patients who were admitted to Renmin Hospital, Hubei University of Medicine between Jan. 2019 and Jun. 2021 were retrospectively selected, and the safety and efficacy of different treatment regimens for PHC were compared and summarized in the present study.

## METHODS

This was a retrospective study. A total of 150 PHC patients who were treated in Renmin Hospital, Hubei University of Medicine between January 2019 and June 2021 were included. They were divided into the control group and the experimental group based on the treatment they received. Patients in the control group received TACE intervention, and those in the experimental group received TACE combined with targeted therapy.

### Ethical Approval:

The study was approved by the Institutional Ethics Committee of Renmin Hospital (No.: syrmyy2023-063; October 11, 2023), and written informed consent was obtained from all participants.

### Inclusion criteria:

- Patients who met PHC diagnostic criteria with no surgical indications;
- With an expected lifespan of ≥three months;
- Had TACE treatment indications and tolerated the treatment;
- With no history of drug allergies;
- Didn’t receive other anti-cancer treatments;
- With ≤three tumor lesions;
- With no other organic lesions;
- And with complete baseline information and follow-up data.

### Exclusion criteria:

- Included patients with mental disorders;
- Coagulation disorders;
- Malignant tum-ors in other sites;
- History of medication use for major organ injuries within one month;
- Severe cardiovascular and cerebrovascular diseases;
- Cognitive dysfunction; contraindications to TACE;
- And esophageal variceal bleeding.

Patients in the control group received TACE treatment, using 10 g lipiodol as the embolic agent. TCE regimen included gemcitabine 1.0-1.6 g/m^2^, pirarubicin 30-50 mg/m^2^, and mitomycin 2-8 mg/m^2^. Patients in the experimental group received TACE combined with targeted therapy. TACE regimen included gemcitabine 1.0-1.6 g/m^2^, pirarubicin 30-50mg/m^2^, and mitomycin 2-8mg/m^2^. On Day three after TACE treatment, 200 mg sorafenib was orally administered once in two days, followed by a treatment-free interval of 3 days, and then proceeded with TACE again. Both groups of patients were evaluated after four weeks of treatment, and those with suboptimal response were repeatedly treated.

### Statistical Analysis:

Data were analyzed using SPSS26.0 software. Enumeration data were presented as frequency, and X^2^ or Fisher’s exact tests were used for comparison between groups. Measurement data were presented as median values, and independent sample test was used for comparison between groups. Survival rate was compared by Log-rank test. Differences with p<0.05 were considered statistically significant.

## RESULTS

One hundred and fifty patients with PHC were included in the study, with 74 in the control group and 76 in the experimental group. No significant difference in baseline information was noted between the two groups (P>0.05) (Table-I).

**Table-I:** Comparison of baseline information.

Baseline information		The control group (n=74)	The experimental group (n=76)	X2/F	P
Sex	Male	39(52.71%)	40(52.63%)	0.000	0.993
	Female	35(47.30%)	36(47.37%)		
Hepatitis B co-infection	yes	30(40.54%)	31(40.79%)	0.001	0.975
	No	44(59.46%)	45(59.21%)		
Combined cirrhosis	Yes	21(28.38%)	23(30.26%)	0.064	0.800
	No	53(71.62%)	53(69.74%)		
Child	A	36(48.65%)	36(47.37%)	0.025	0.875
	B	38(51.35%)	40(52.63%)		
ECOG-PS	0-1	38(51.35%)	39(51.32%)	0.000	0.997
	2	36(48.65%)	37(48.69%)		
Tumor diameter	<5cm	35(47.30%)	36(47.37%)	0.000	0.993
	≥5cm	39(52.71%)	40(52.63%)		
TNM	Stage III	38(51.35%)	39(51.32%)	0.000	0.997
	Stage IV	36(48.65%)	37(48.69%)		
Age		52.99±6.75	52.87±6.97	0.105	0.916
Number of TACE		2.35±0.48	2.25±0.44	1.353	0.178

Efficacy of the two groups was evaluated using RECIST, and the response rate and DCR of the experimental group were significantly increased compared with those of the control group (P<0.05) (Table-II). All patients were followed up for three years, and significant differences in the one and three years survival and relapse rates were seen between the two groups (P<0.05) (Table-III).

**Table-II:** Comparison of short-term efficacy

Efficacy	The control group(n=74)	The experimental group(n=76)	X^2^	P
CR	0(0.00%)	0(0.00%)	-	-
PR	31(41.89%)	59(77.63%)	-	-
SD	25(33.78%)	10(13.16%)	-	-
PD	18(24.32%)	7(9.21%)	-	-
ORR (%)	31 (41.89%)	59(77.63%)	19.955	<0.001
DCR (%)	56(75.68%)	69(90.79%)	6.116	0.013

**Table-III:** Comparison of long-term efficacy.

Groups	1 year		3 year	

	Relapse rate	Survival rate	Relapse rate	Survival rate
The control group(n=74)	41(55.41%)	31(41.89%)	73(98.65%)	7(9.46%)
The experimental group(n=76)	29(38.16%)	48(63.16%)	47(61.84%)	18(23.68%)
*X* ^2^	4.481	6.802	31.746	5.462
*P*	0.034	0.009	<0.001	0.019

There were no significant differences in AFP, VEGF, ALT, AST, TBIL, and GGT between the two groups of patients before treatment. After treatment, the levels of AFP, VEGF, ALT, and AST in the experimental group were significantly decreased compared with those in the control group (p<0.05), and no significant differences in TBIL and GGT were noted between the two groups (p>0.05) (Table-IV).

**Table-IV:** Comparison of biochemical parameters.

Biochemical parameters		The control group(n=74)	The experimental group(n=76)	t	P
AFP (ng/mL)	One day before treatment	975.26±192.34	972.36±193.24	0.092	0.927
	One month after treatment	625.75±113.75	518.63±106.98	5.943	<0.001
VEGF(Pg/mL)	One day before treatment	262.75±8.95	261.98±9.26	0.514	0.608
	One month after treatment	226.87±8.79	177.26±8.26	35.621	<0.001
ALT(U/L)	One day before treatment	55.36±7.54	55.76±5.79	0.363	0.717
	One month after treatment	58.76±8.94	44.38±9.12	9.746	<0.001
AST(U/L)	One day before treatment	86.25±8.94	85.94±9.12	0.209	0.835
	One month after treatment	88.94±8.75	61.25±8.73	19.394	<0.001
TBIL (umol/L)	One day before treatment	14.21±1.72	14.32±1.28	0.441	0.660
	One month after treatment	14.98±3.75	14.15±1.97	1.704	0.090
GGT(U/L)	One day before treatment	122.35±8.96	122.75±8.68	0.972	0.333
	One month after treatment	127.12±9.23	126.21±8.95	0.614	0.540

During the treatment period, both groups of patients experienced complications such as decreased WBC, decreased N, decreased Hb, bleeding, diarrhea, nausea and voting, and fever. The incidence and severity of complications of diarrhea, nausea and voting, and fever in the experimental group were significantly decreased compared those in the control group, and the differences were statistically significant (p<0.05) (Table-V).

**Table-V:** Adverse reactions.

Adverse reactions	The control group(N=74)					The experimental group(N=76)					Z	P

	0	I	II	III	IV	0	I	II	III	IV		
WBC↓	61	7	4	1	1	65	6	3	1	1	0.320	0.988
N↓	32	22	18	1	1	45	20	10	1	0	5.550	0.235
Hb↓	34	19	18	2	1	45	16	14	1	0	3.596	0.463
Bleeding	70	2	2	0	0	75	1	0	0	0	2.480	0.289
Diarrhea	41	12	13	6	2	52	19	5	0	0	14.413	0.006
nausea and vomiting	38	19	15	1	1	59	9	8	0	0	12.224	0.016
fever	37	19	15	2	1	61	9	6	0	0	16.282	0.003

## DISCUSSION

TACE can cause liver damage and even liver failure. In this study, biochemical indicators such as AFP, VEGF, ALT, AST, TBIL, and GGT were used to reflect disease control and the degree of liver function damage. The results showed that there was no significant difference in TBIL and GGT indicators between the two groups of patients after treatment (p>0.05), while the levels of AFP, VEGF, ALT, and AST in the observation group were significantly lower than those in the control group (p<0.05), indicating that TACE combined with targeted drugs can significantly reduce the level of tumor markers in PHC and effectively improve liver function in patients.

PHC has the characteristics of occult onset, high incidence rate, high mortality, and short estimated survival time.^7^ Most patients are in the middle and late stages of missing the best treatment opportunity when diagnosed. TACE is an effective treatment plan for the treatment of late PHC. TACE can selectively block tumor blood supply vessels, make the tumor focus ischemic and necrotic, shrink the focus, and control tumor progress.^8^ However, in the actual clinical application process, TACE treatment may encounter a series of complications, leading to the inability to continue treatment, and single TACE treatment has limitations and cannot meet the treatment needs of PHC.^9^ Targeted therapy is one of the main treatment options for PHC, which can block the signaling pathways of tumor cell growth and proliferation, achieve the goal of killing tumor cells and inhibiting tumor proliferation. Sorafenib is a representative drug for targeted therapy of PHC, and it is also the earliest molecular targeted drug used for anti-tumor treatment of PHC. This drug can effectively prolong the overall survival of PHC patients, delay their progression time, and has high tolerance.^10^

Previous studies have found that the application of combination therapy is a key direction and content of PHC treatment research.^11^-^16^ Meyer et al.^17^ pointed out in the phase three trial of sorafenib combined with TACE for HCC that DEB-TACE combined with sorafenib cannot improve progression free survival in HCC patients in Europe. Kimura et al.^18^ pointed out that the five-year survival prognosis of patients with advanced liver cancer treated with sorafenib combined with TACE is 36.3%, and the five-year survival prognosis of patients with advanced liver cancer treated with sorafenib alone is 7.7%. Kudo et al.^19^ pointed out that the combination of TACE and sorafenib can prolong the median FPS of HCC patients to 25.2 months, significantly higher than the 13.5 months of the single TACE group. TACE combined with sorafenib significantly increases the PFS of HCC patients, and there are no unexpected toxic reactions during the treatment process, indicating high safety. Varghese et al.^20^ pointed out that in BCLC phase B and phase C HCC interventions, the combination of sorafenib and TACE can extend the overall survival from four months to nine months. The survival benefits and tumor intervention efficacy of the sorafenib combined TACE regimen are significantly better than those of a single TACE or sorafenib intervention regimen. The results of this study showed that the disease remission and control rates of patients in the study group treated with TACE combined with targeted therapy were significantly higher than those in the control group treated with TACE alone (77.63% vs 41.89%, 90.79% vs 75.68%), with statistical significance (P<0.05). The results of this study also showed that the 1-year and 3-year survival rates of patients in the observation group were significantly higher than those in the control group (63.16% vs 41.89%, 23.68% vs 9.46%), while the recurrence rate was lower than that in the control group (39.47% vs 52.71%, 61.84% vs 98.65%), with statistical significance (P<0.05), indicating that TACE combined with targeted drugs can improve PH. The clinical benefits of C patients are consistent with the previous research results mentioned above.

Regarding the safety of TACE combined with targeted drugs in the treatment of PHC, the results of this study showed that during the treatment period, both groups experienced varying degrees of complications. The incidence and severity of complications such as Diarrhoea, nausea and vomiting, and generate heat in the study group were significantly lower than those in the control group, and the difference was statistically significant (p<0.05), indicating that the combined regimen is safe.

### Limitations:

It includes as small sample size, small course of treatment, and short follow-up time. It still needs further clinical research to observe the long-term clinical effect of TACE combined with targeted therapy in the treatment of PHC, so as to apply a better treatment modality to patients in need.

## CONCLUSIONS

The results of the present study confirmed that the clinical efficacy and safety of TACE combined with sorafenib targeted therapy are excellent in the treatment of PHC, and can be investigated in future studies for PHC intervention.

### Authors’ Contributions:

**XW and DZ** designed this study, prepared this manuscript are responsible and accountable for the accuracy and integrity of the work.

**KL, PG and ZL** performed the statistical analysis participated in its design. All authors have read and approved the final manuscript.
